# WGCNA, LASSO and SVM Algorithm Revealed RAC1 Correlated M0 Macrophage and the Risk Score to Predict the Survival of Hepatocellular Carcinoma Patients

**DOI:** 10.3389/fgene.2021.730920

**Published:** 2022-03-18

**Authors:** Ji-An You, Yuhan Gong, Yongzhe Wu, Libo Jin, Qingjia Chi, Da Sun

**Affiliations:** ^1^ College of Technology, Hubei Engineering University, Xiaogan, China; ^2^ Department of Geotechnical Engineering, Wuhan University of Technology, Wuhan, China; ^3^ Department of Mechanics and Engineering Structure, Wuhan University of Technology, Wuhan, China; ^4^ Institute of Life Sciences and Biomedical Collaborative Innovation Center of Zhejiang Province, Wenzhou University, Wenzhou, China

**Keywords:** hepatocellular carcinoma, RAC1, macrophages, gene signature, independent prognostic markers

## Abstract

**Background:** RAC1 is involved in the progression of HCC as a regulator, but its prognostic performance and the imbalance of immune cell infiltration mediated by it are still unclear. We aim to explore the prognostic and immune properties of RAC1 in HCC.

**Methods:** We separately downloaded the data related to HCC from the Cancer Genome Atlas (TCGA) and GEO database. CIBERSORT deconvolution algorithm, weighted gene co-expression network analysis (WGCNA) and LASSO algorithm participate in identifying IRGs and the construction of prognostic signatures.

**Results:** The study discovered that RAC1 expression was linked to the severity of HCC lesions, and that its high expression was linked to a poor prognosis. Cox analysis confirmed that RAC1 is a clinically independent prognostic marker. M0, M1 and M2 macrophages’ abundance are significantly different in HCC. We found 828 IRGs related to macrophage infiltration, and established a novel 11-gene signature with excellent prognostic performance. RAC1-based risk score and M0 macrophage has a good ability to predict overall survival.

**Conclusion:** The immune state of irregular macrophage infiltration may be one of the precursors to carcinogenesis. The RAC1 correlated with M0 macrophage and the risk score to show a good performance to predict the survival of HCC patients.

## Introduction

Hepatocellular carcinoma (HCC) is a serious malignant disease with a high recurrence rate. Although many patients have been diagnosed and treated early in the disease (Shiani et al., 2017). The prognosis is even less optimistic for patients who have entered the middle and advanced stages. If cancer spreads to the surrounding lymph nodes, the 5-year survival rate for the patient is only 11%. When cancer spreads to other organs, the 5-year survival rate is only 3% (Anwanwan et al., 2020). At present, drugs that inhibit angiogenesis dominate. However, immunotherapy is expected to become one of the most effective therapy for liver cancer within 5 years (Xie et al., 2018). However, immunotherapy has the drawbacks of metastasis, recurrence, and the use of a small number of patients (Jiang et al., 2019). Therefore, new therapies closely related to treatment must be developed.

Ras-related C3 botulism substrate 1 (RAC1) belongs to the RAC subfamily and is among the main members of the Rho family (Parri and Chiarugi, 2010). It is an important signal transduction molecule that changes the cytoskeleton assembly, regulates cell migration, regulates gene transcription and other biological activities (de Conti et al., 2020). It is also involved in regulating malignant phenotype, proliferation, apoptosis, tumor angiogenesis, invasion and metastasis of tumor cells (Maldonado and Dharmawardhane, 2018). Studies have shown that the expression of RAC1 is significantly increased in some malignant tumors, including gastric cancer (Wu et al., 2014), non-small cell lung cancer (Zhou et al., 2016), breast cancer (Tian et al., 2018), etc. At the same time, its high expression is related to the degree of tumor cell differentiation, invasion and metastasis. However, the prognostic performance and clinical landscape of RAC1 in liver cancer are rarely reported. Therefore, exploring the expression and prognostic characteristics of RAC1 is critical for new treatments of liver cancer.

Liver cancer is mainly immunogenic cancer caused by chronic inflammation. The tumor microenvironment’s imbalance is a typical feature of liver cancer (Yang et al., 2019). Immunoregulation of tumor occured in the progress of tumor development, including gene alteration, cell proliferation, anti-apoptosis, and the degradation of tumor cell genome stability (Gonzalez et al., 2018). Currently, the function of immune infiltration in liver cancer remains unexplored. Similarly, the function of different immune cells in the local liver cancer microenvironment is constantly being investigated. HCC cells are killed by CD+ tumor-infiltrating lymphocytes (Ikeguchi et al., 2004). Activated macrophages facilitate HCC progression. The presence of mature dendritic cells decreases the recurrence and metastasis risk after liver surgery (Lin et al., 2006). In addition, immunotherapy for programmed death 1 (PD-1) and programmed death ligand 1 (PD-L1) has been used to treat liver cancer successfully (Shi et al., 2011). As a result, elucidating the features of the immune pattern triggered by RAC1 is critical for liver cancer immunotherapy and prognosis prediction.

In the study, we screened out the RACI gene through preliminary literature search and experiments to explore its prognostic performance in liver cancer. First, we used TCGA data and GSE76427 to evaluate the prognostic value of RAC1 in liver cancer. Subsequently, a co-expression network of genes was constructed through WGCNA with differentially infiltrated immune cells as a clinical trait. Correspondingly, an immune-related gene signature was constructed through LASSO algorithm.

## Materials and Methods

### Data Collection

We downloaded HCC mRNA-seq and clinical information from the TCGA database, including 371 tumor and 50 normal samples. The expression of genes is all logarithmic. At the same time, the expression data of GSE76427 and the corresponding clinical data from the GEO database (https://www.ncbi.nlm.nih.gov/geo) served as validation data set. GSE76427 contains 115 tumor tissues and 52 adjacent tissues. The non-zero survival time was retained for clinical analysis in the TCGA and GSE76427 data sets.

Clinical evaluation of RAC1 integrates the expression data of RAC1 and the survival data of the patients to evaluate the prognostic value. We applied *t* test to infer the expression changes of RAC1 in various stages of HCC. Then, we analyzed the correlation between RAC1 and the overall survival (OS) through the Kaplan-Meier (K-M) curve. The log-rank test was used to compare the prognostic differences between the groups. The diagnostic capability of RAC1 is measured by the area under the time-dependent receiver operating characteristics (tROC) curve (AUC).

### Cox Regression Model

The Cox regression model establishes the relationship between the survival rate of patients and several variables and evaluates the effects of these factors on survival time. When *p* < 0.05, the impact on survival time is statistically significant, identifying the expression profiles of 338 immune-related genes. The survival analysis aims to study the relationship between the variable X and the survival function (cumulative survival rate) S (t, X). X = (X_1,…, X_m) is a vector, and S (t, X) is affected by many factors. The traditional method considers the regression equation, that is, the influence of variables X_i on S (t, X). However, the data in the survival analysis contains censored data, and the time t usually does not meet the requirements of normal distribution and homogeneity of variance. These reasons make it difficult to study the above-mentioned relationship with the general regression method. Therefore, we use the Cox regression model as a specific tool (Goerdten et al., 2020). The Cox regression is implemented through the “survival” package in R.

### Support Vector Machine

Support vector machine (SVM) is a class of generalized linear classifiers that classify data binary in a supervised learning manner. Its decision boundary is the maximum margin hyperplane that is solved for learning samples (Huang et al., 2018). CIBERSORT performed deconvolution based on ν’s support vector machine (ν-SVR) method, a support vector machine (SVM) optimization method for binary classification problems. Based on deconvolution and linear support vector regression principles, CIBERSORT evaluates 22 immune cell molecular subtypes and uses v-SVR to estimate immune scores based on the expression matrix of IRGs. Therefore, the SVM algorithm is used in this paper through CIBERSORT.

### Immune Cell Infiltration Analysis

CIBERSORT is a deconvolution tool that links immune cell infiltration to gene expression. It includes the LM22 gene signature file, which contains 547 genes used to identify 22 immune cell subtypes. We uploaded the mRNA expression data to the CIBERSORT portal (https://CIBERSORT.stanford.edu/). The gene expression data can then be standardized by running 1,000 permutations through the default feature matrix. Finally, we applied WilCoxon test to determine the differentially infiltrated immune cells in tumors. The deconvolution algorithm CIBERSORT (Newman et al., 2015) is used to determine the immune-related features from 365 labeled immune genes, and to quantify the relative score of each immune cell type. The method is used to evaluate the relative proportions of 22 tumor-infiltrating immune cell profiles based on expression files, including B cells, T cells, natural killer cells, macrophages, dendritic cells, and bone marrow subpopulations. And we used gene expression data to estimate the abundance of member cell types in the mixed cell population. Monte Carlo sampling is used to obtain the *p*-value for each deconvolution sample through CIBERSORT algorithm. The number of permutations is set to 1,000, and *p* < 0.05 is considered significant. The immune cell matrix of each sample was obtained in the transcriptome data through the CIBERSORT algorithm.

### WGCNA Analysis

The R platform’s “limma” software package is used to find genes that are differentially expressed in the high expression community vs. the low expression progenitor of RAC1 (*p* 0.05 and (log 2 FC) > 1). Then we built a co-expression network of differentially expressed genes using the R package “WGCNA.” WCGNA clusters IRGs with similar expression patterns to construct a scale-free gene co-expression network and analyzes the correlation between modules and specific phenotypes. The important IRG modules are identified according to the module-IRG, module-module correlation. WGCNA is a robust algorithm highlighted by the modular clustering of genes and the association analysis between the modules and clinical traits.

First, we used a hierarchical cluster analysis on the expression profile.

$$S=\left[S_{XY}\right]=\left[\left|cor\left(X,Y\right)\right|\right]$$

To create the adjacency matrix A, calculate the correlation index an XY of any gene pair using the square of the correlation coefficient S XY.

$$A=\left[a_{XY}\right]=\left[power\left(S_{XY},\beta \right)\right]=\left[{\left|S_{XY}\right|}^{\beta }\right]$$

Then, a topological overlap matrix (TOM) is constructed,

$$TOM=\left[\omega_{XY}\right]=\left[\frac{l_{XY}+a_{XY}}{\min\left\{k_{X},k_{\text{Y}}\right\}+1-a_{XY}}\right]$$

### The Construction of Risk Prediction Signature

The R software package “survival v3.1-8” was used to perform univariate Cox regression on the immune module genes in the TCGA cohort. *p* < 0.05 is used to screen for genes significantly related to the overall survival rate of patients. We used the R software package “glmnet v3.0-2” to perform LASSO analysis to optimize the results further. LASSO appeared as a constraint on the objective function.

$$\underset{\omega }{\min}\sum_{j=1}^{m}{\left(y_{j}-\sum_{i=1}^{n}x_{ji}\omega_{i}\right)}^{2},s.t.\sum_{i=1}^{n}\left|\omega_{i}\right|\leq \lambda$$

The risk score (RS) referred to

$$RS=\sum_{i=1}^{n}\beta_{i}\times Exp_{i}$$

where *n* denoted the prognostic signature genes. The LASSO coefficient of a gene is represented by *i*. The expression of a gene is represented by *Exp*_*i*_.

LASSO COX performs collinearity processing on the filtered genes. Thus, the algorithm has advantages in processing high-dimensional data. The screening performed 10-fold cross-validation, and the survival status and time were used as dependent variables to analyze the influence of multiple IRGs on the dependent variables. Finally, the LASSO coefficient is determined when the lambda is the minimum, forming the IRG signatures.

### Analytical Statistics

The “R v3.6.1″ framework is used for all statistical analysis R packages. Statistical significance was described as a *p*-value of less than 0.05.

## Results

### Clinical Manifestations of RAC1

**FIGURE 1:**
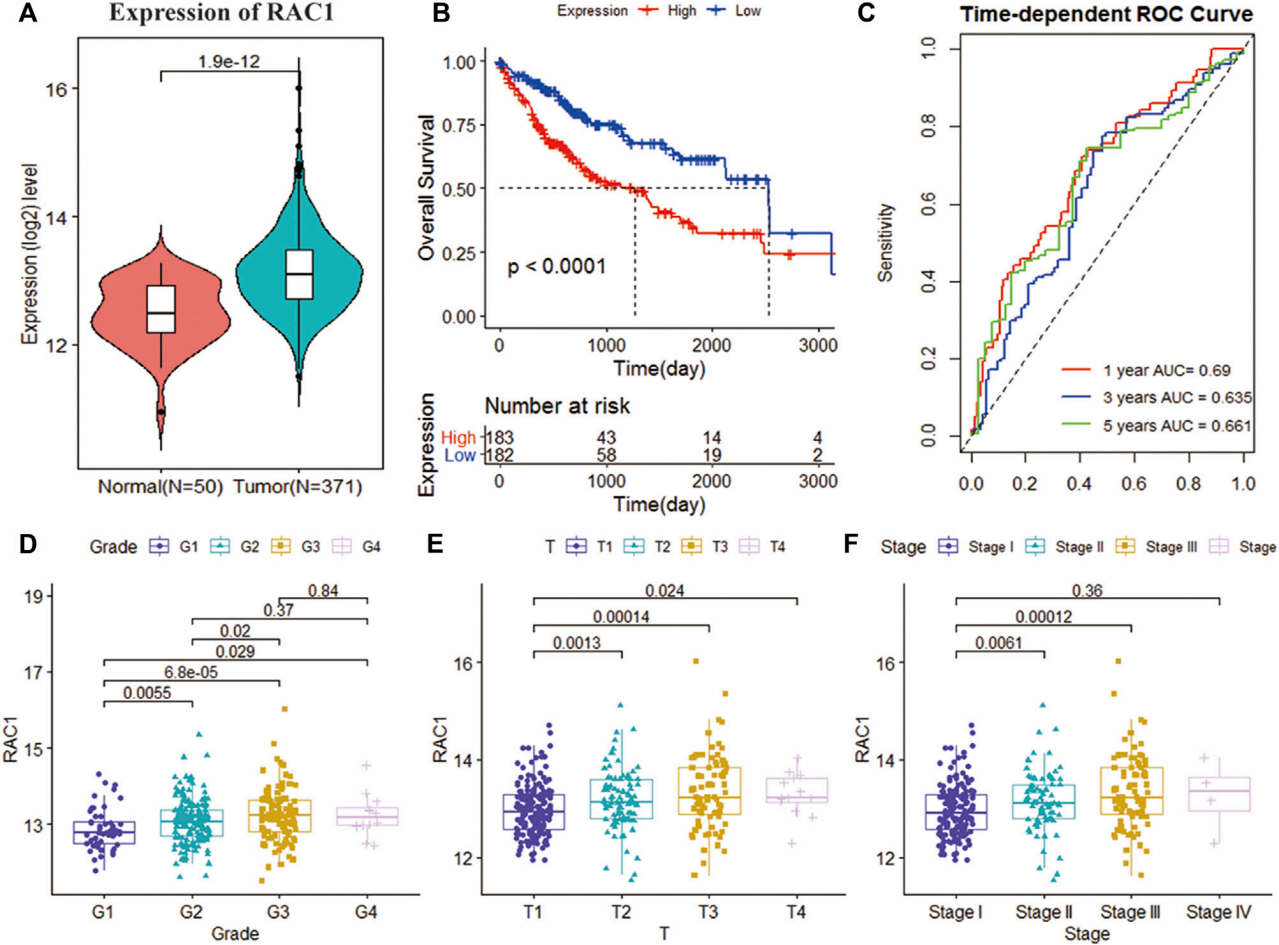
Analysis of the clinical performance of RAC1 in the TCGA-LIHC cohort. **(A)** Violin chart showing the relative expression of RAC1 in normal and tumor samples; **(B)** K-M curve of the high- and low- RAC1 expression group of the TCGA cohort; **(C)** ROC curve of the TCGA cohort, for 1, 3, and 5 years; **(D–F)** shows a box plot of the relative expression of RAC1 in different strata of disease stage and tumor grade. The central marker is the median, and the *t*-test is used to estimate the importance of differences in gene expression between the two groups.

The TCGA cohort collected the pathological characteristics of 365 patients to evaluate the clinical manifestations. We studied the gene expression profile and clinical information of 115 patients with a non-zero survival time of GSE76427. RAC1 expression was substantially higher in tumor tissues than in normal ones (*p* = 1.9e-12; Figure 1A). Kaplan-Meier results showed that the increase in RAC1 expression was closely linked to the poor patient survival rate (*p* < 0.001, Figure 1B). In addition, RAC1 has excellent capabilities in HCC (1-, 3-, 5-year AUC = 0.690, 0.635, 0.661; Figure 1C). The GSE76427 cohort also confirmed that RAC1 has a good prognostic ability (Figure 1). Interestingly, RAC1 expression was significantly correlated with the grade, T, and stage (Figures 1D–F).

### Independent Prognostic Landscape of RAC1

**FIGURE 2:**
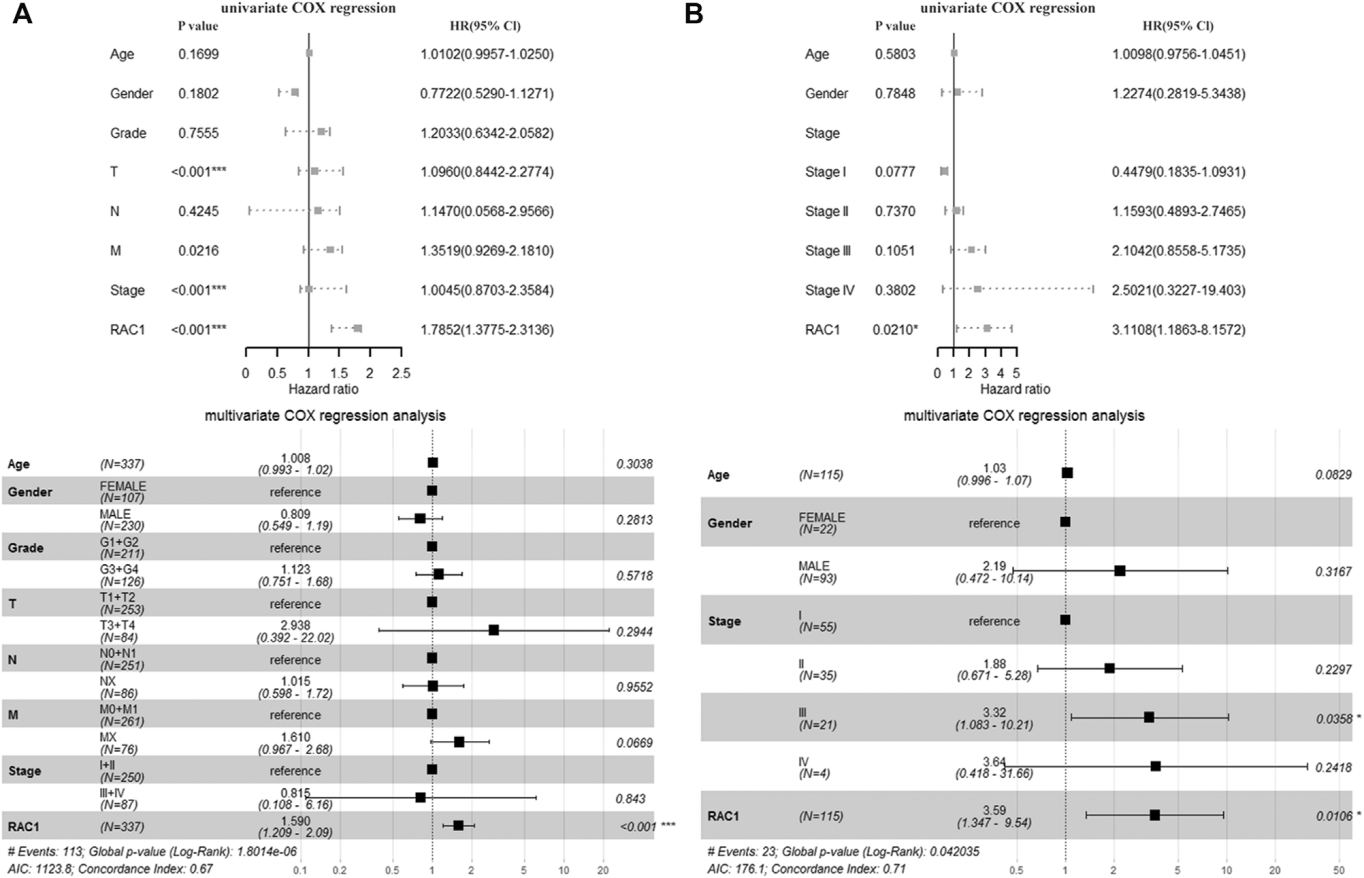
The prognostic significance of RAC1. **(A)** In the TCGA cohort, T, Stage and RAC1 were significantly related to the overall survival rate of patients. The multivariate Cox of the TCGA cohort. The regression found that RAC1 is an independent prognostic marker of HCC; **(B)** In the GSE76427 cohort, we found that RAC1 significantly affected the survival of patients; Multivariate Cox regression of the GSE76427 cohort indicated that stage III and RAC1 are risk factors of HCC.

In the TCGA cohort, T (HR = 1.0960, 95% CI = 0.8442–2.2774, *p* < 0.001), Stage (HR = 1.0045, 95% CI = 0.8703\−2.3584, *p* < 0.001) and RAC1 (HR = 1.7852, 95% CI = 1.3775–2.3136, *p* < 0.001) was significantly correlated with the overall survival of the patient. Multivariate Cox regression found RAC1 (HR = 1.590, 95% CI = 1.209–2.09, *p* < 0.001) was an independent progostic marker of HCC (Figure 2A). In the GSE76427 cohort, we found that RAC1 (HR = 3.1108, 95% CI = 1.1863–8.1572, *p* = 0.0210) significantly affected the survival of patients. In addition, multivariate Cox regression found that Stage III (HR = 3.32, 95% CI = 1.083–10.21, *p* = 0.0358) and RAC1 (HR = 3.590, 95% CI = 1.347–9.54, *p* = 0.0106) are risk factors for HCC (Figure 2B).

### Tumor Immune Microenvironment Fluctuation

**FIGURE 3:**
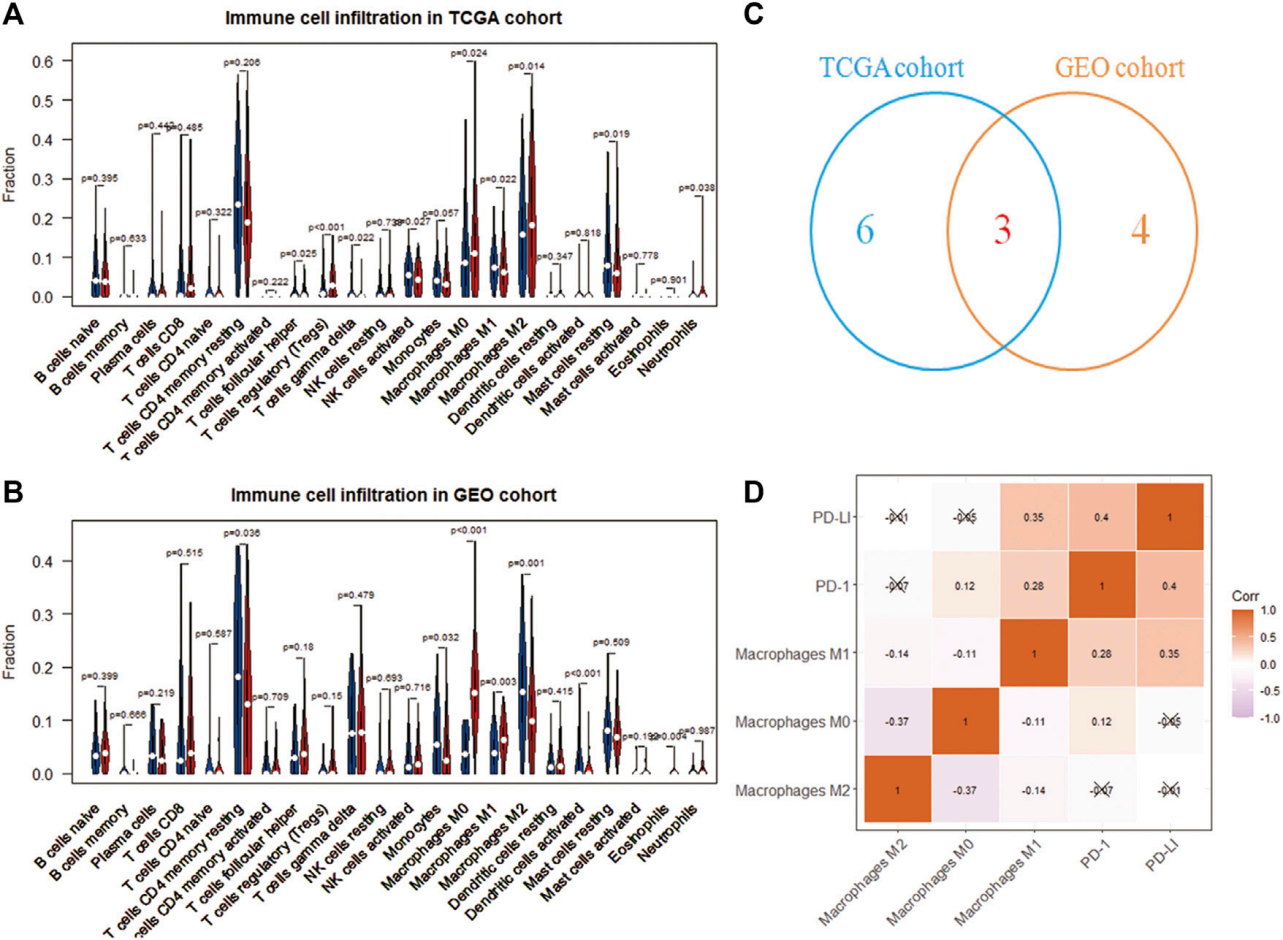
Immune cell analysis driven by RAC1. **(A,B)** Differences in the infiltration of 22 immune cells in the high- and low-expression of RAC1; **(C)** Venn diagram identify three types of immune cells; **(D)** Correlation analysis between RAC1 and immune cells and immune checkpoint inhibitors (PD-1 and PD-L1).

The CIBERSORT deconvolution algorithm obtained the infiltration level of 22 immune cells. Grouping 365 tumor samples with high and low expression of RAC1, we found differences in the infiltration of nine immune cells in the TCGA cohort (Figure 3A). In the GSE76427 cohort, we found seven differences in immune cell fluctuations (Figure 3B). Macrophages M0, M1, and M2 were identified by Wayne analysis (Figure 3C). We speculate that RAC1 mediates macrophages to play a critical role in the development of HCC. The expression of immune checkpoint inhibitors (PD-1 and PD-L1) was significantly correlated with macrophage M1 (Figure 3D).

### WGCNA Recognizes Immune Module Genes

**FIGURE 4:**
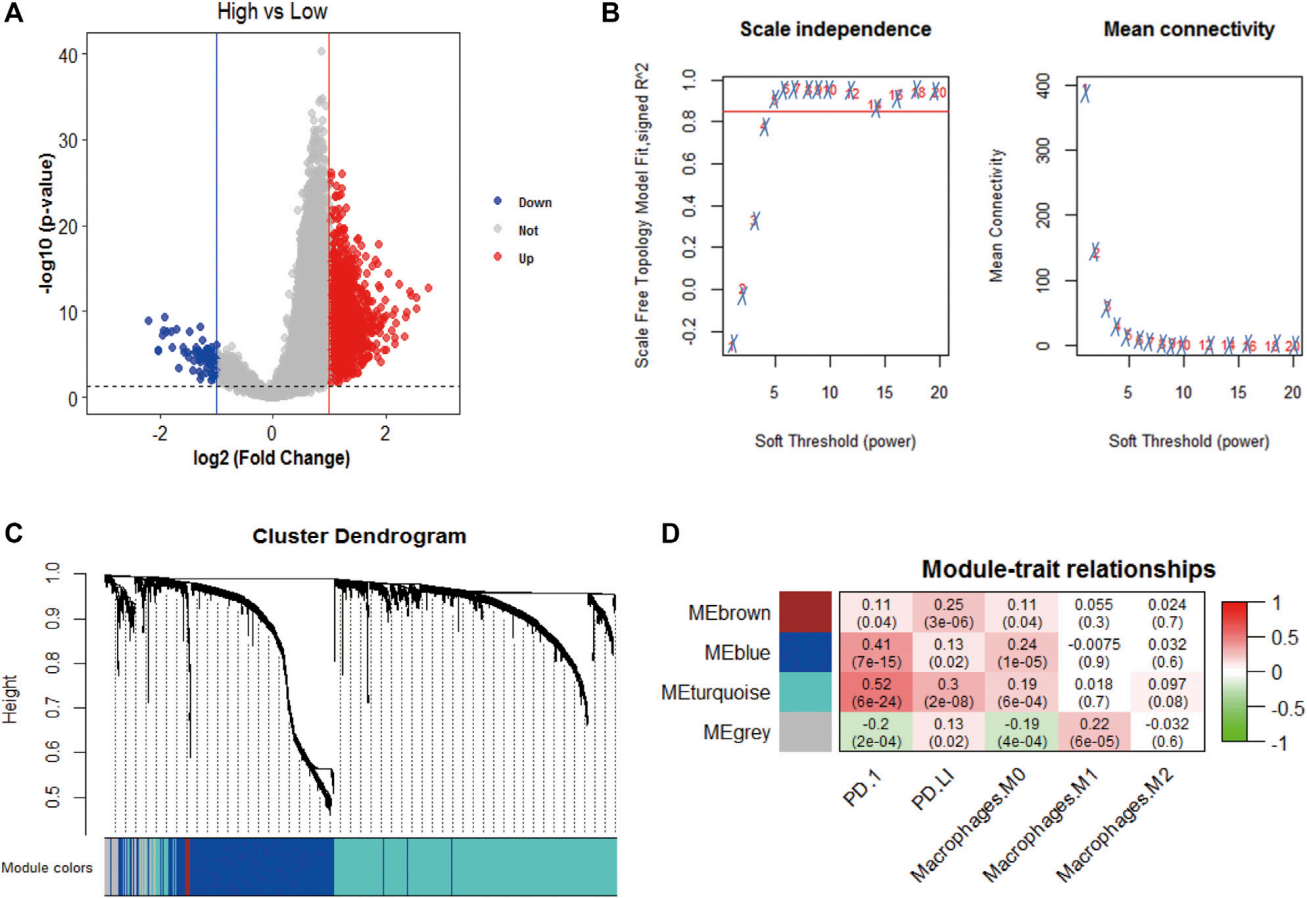
WGCNA analysis. **(A)** Volcano plot analysis of differentially expressed genes, 80 were upregulated and 1,337 were downregulated; **(B)** Unscaled fitting index of soft threshold power. The soft threshold power of WGCNA is determined based on the scale-free fitting index R2. The left panel shows the relationship between the soft threshold and R2. The panel on the right shows the relationship between soft threshold and average connectivity. **(C)** Dendrogram of clusters of differentially expressed genes based on different metrics. Each branch in the figure represents a gene, and each color below represents a co-expression module. **(D)** A heat map showing the correlation between gene modules and clinical features. The turquoise module contains 836 immune-related genes. The correlation coefficient of each cell represents the correlation between gene modules and clinical features, and the size decreases from red to green. The red module has the highest positive correlation with survival and the green module has the highest negative correlation with survival.

We obtained 1,417 differentially expressed genes (DEGs), of which 80 were upregulated and 1,337 were downregulated (Figure 4A). WGCNA analysis was performed based on the mRNA expression data of 1,417 DEGs. The soft threshold is selected as 5 to conform to the scale-free network rule through hierarchical clustering (Figure 4B). A total of four gene modules were identified using the clustering criteria of minModuleSize = 30 and mergeCutH8 = 0.25 (Figure 4C). TOM is used to describe the pair-wise relationship between genes. In the topological overlap matrix (TOM), each row and each column corresponds to a gene. Light colors indicate low topological overlap, and gradually darker ones indicate higher topological overlap. The dark squares along the diagonal correspond to the modules. The gene tree diagram and module allocation are shown on the left and top. The four gene modules are gray, turquoise, yellow, blue, and brown modules. The correlation analysis between the module and the clinical phenotype showed that the turquoise module is related to PD-1 (cor = 0.52, *p* = 6e-24), PD-L1 (cor = 0.3, *p* = 2e-08), and macrophages M0 (cor = 0.19, p = 6e-04) is significantly correlated (Figure 4D). Therefore, the turquoise module (*n* = 838) was identified as immune-related genes (IRGs) for subsequent analysis.

### Construction of Immune-Related Genes Signature

**FIGURE 5:**
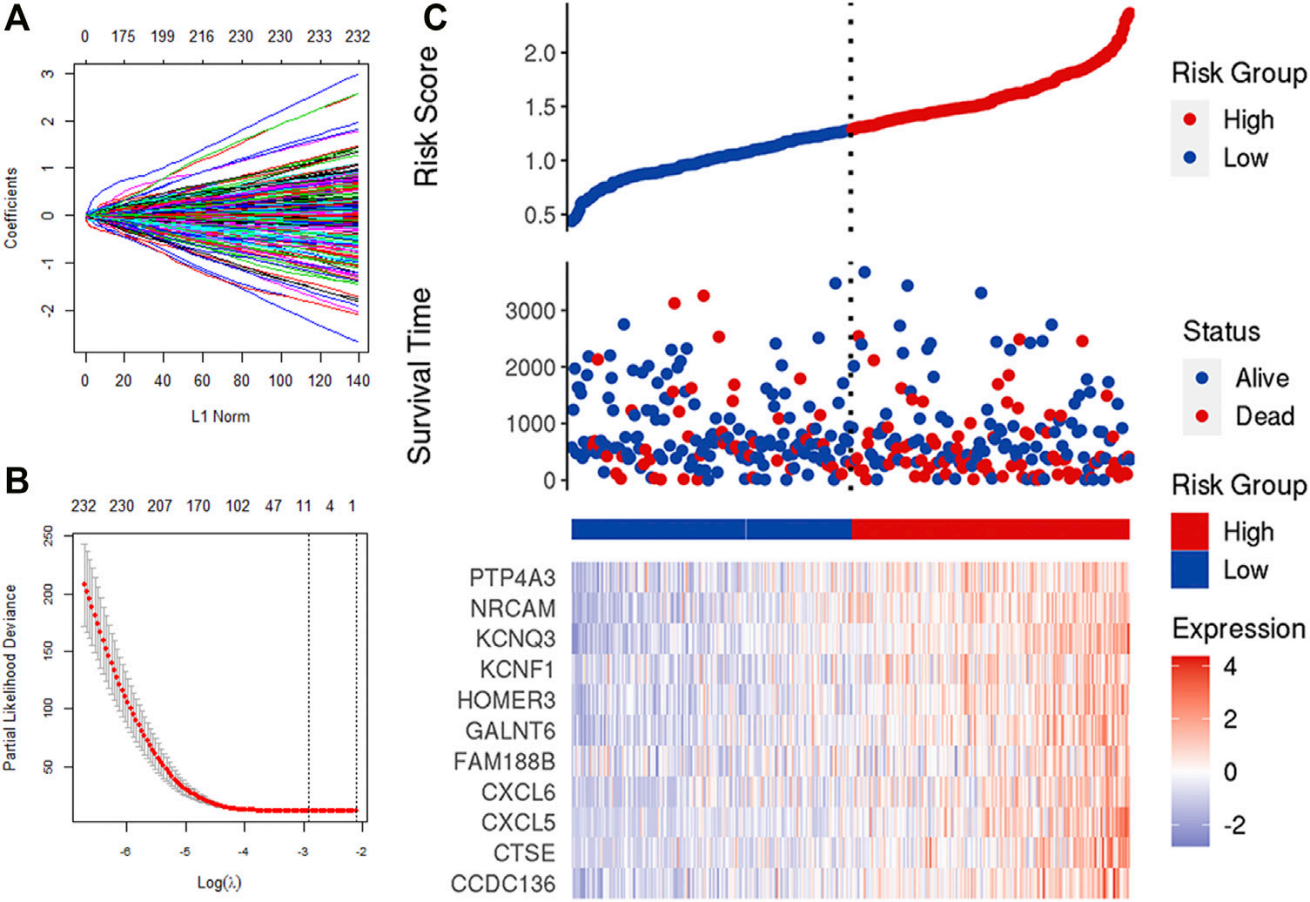
LASSO analysis. **(A,B)** Determine the number of genetic screenings. **(C)** Analysis of prognostic risk model.

We obtained 388 IRGs that significantly correlated with the survival through univariate Cox analysis. Subsequently, LASSO Cox analysis was used to optimize the results further. We finally obtained 11 genes (CCDC136, CTSE, CXCL5, CXCL6, FAM188B, GALNT6, HOMER3, KCNF1, KCNQ3, NRCAM, PTP4A3), which are significantly related to the survival rate of patients (Figures 5A–C). Figure 5 shows the identification of immune-related genes. LASSO COX determines the number of factors by introducing shrinkage penalties and limiting the coefficients. With the continuous selection and simulation of the number of features, the best model and the simplest model are finally obtained. Subsequently, we further calculate the final score of the model. The final risk score is obtained by multiplying the expression of each gene with its corresponding coefficient and adding them together. And, with the median risk score of all patients as the critical value, the patients were divided into high-risk and low-risk two groups.

The risk scoring formula is as follows:

$$\begin{array}{c} \text{RS }=\text{CCDC}136\text{*0}\text{.}0228+\text{CTSE*0}\text{.}0176+\text{CXCL}5\text{*0}\text{.}0244\\ +\text{CXCL}6\text{*0}\text{.}006+\text{FAM}188\text{B*0}\text{.}0016+\text{GALNT}6\text{*0}\text{.}0326\\ +\text{HOMER}3\text{*0}\text{.}001+\text{KCNF}1\text{*0}\text{.}0056+\text{KCNQ}3\text{*0}\text{.}0916\\ +\text{NRCAM*0}\text{.}0298+\text{PTP}4\text{A}3\text{*0}\text{.}0247 \end{array}$$

**FIGURE 6:**
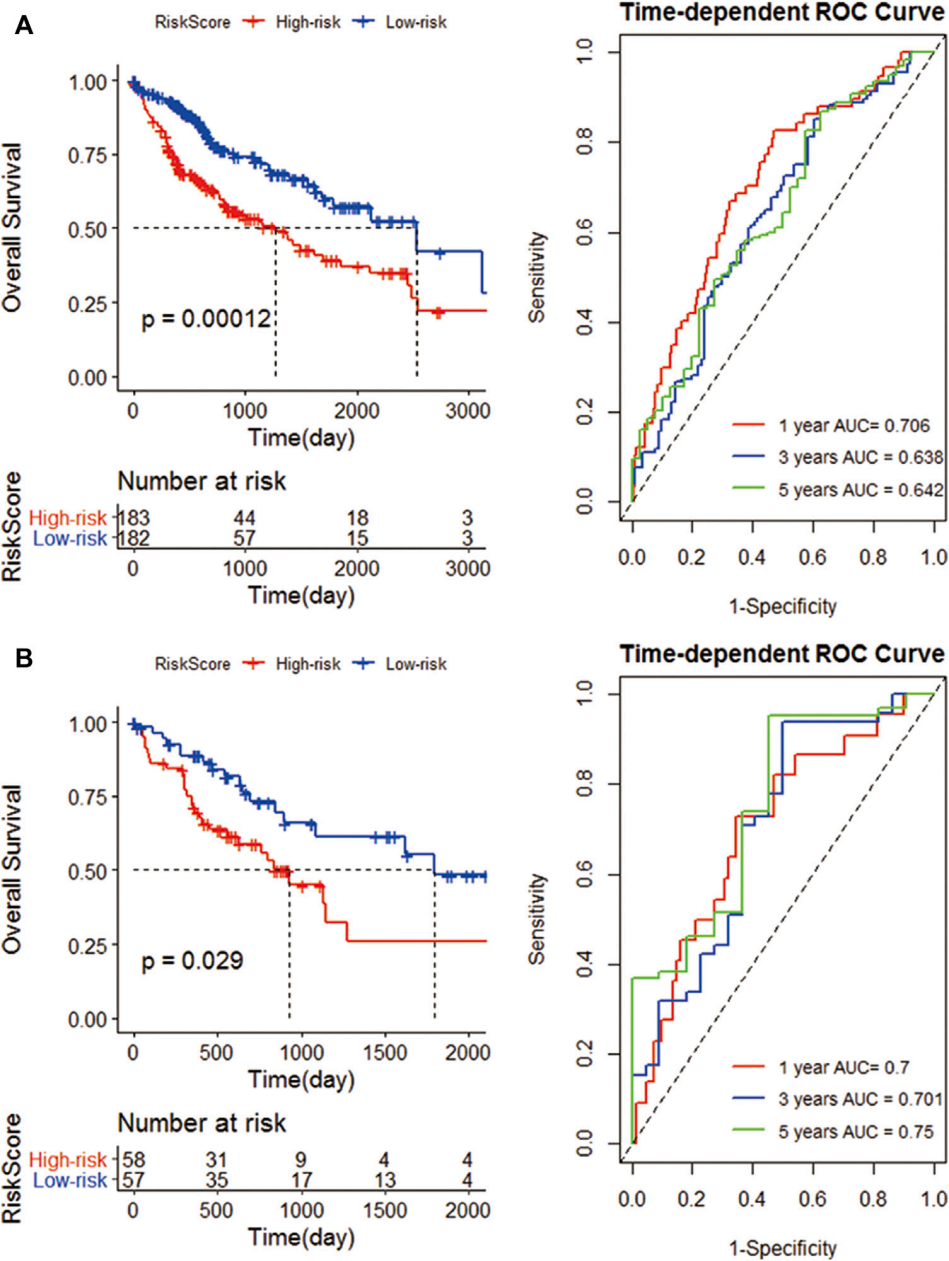
Construction of an IRG signature. **(A)** K-M curves of the high- and low-risk groups of the TCGA cohort. Compared with high-risk group, low-risk significantly improve the prognosis of patients; ROC curves, 1, 3, and 5 years are 0.706, 0.638 and 0.642 respectively; **(B)** The K-M curves of the high and low risk groups of the GEO cohort; and the 1-year, 3-year, and 5-year survival rates.

Patients were divided into low- and high-risk groups (152 vs. 153) according to the risk score in the TCGA cohort. Compared with high risk, low risk can significantly improve the prognosis of patients (*p* = 0.00012). The 1-, 3-, and 5-year AUC (0.706, 0.638, and 0.642) confirmed the signature’s good prognostic performance (Figure 6A). In addition, the K-M analysis (*p* = 0.029) and ROC analysis also confirmed the above results in the validation cohort. The 1-, 3-, and 5-year survival rates of the risk prognostic feature prediction validation cohort were 0.700, 0.701, and 0.750, respectively (Figure 6B).

### Selection of Prognostic Factors

**FIGURE 7:**
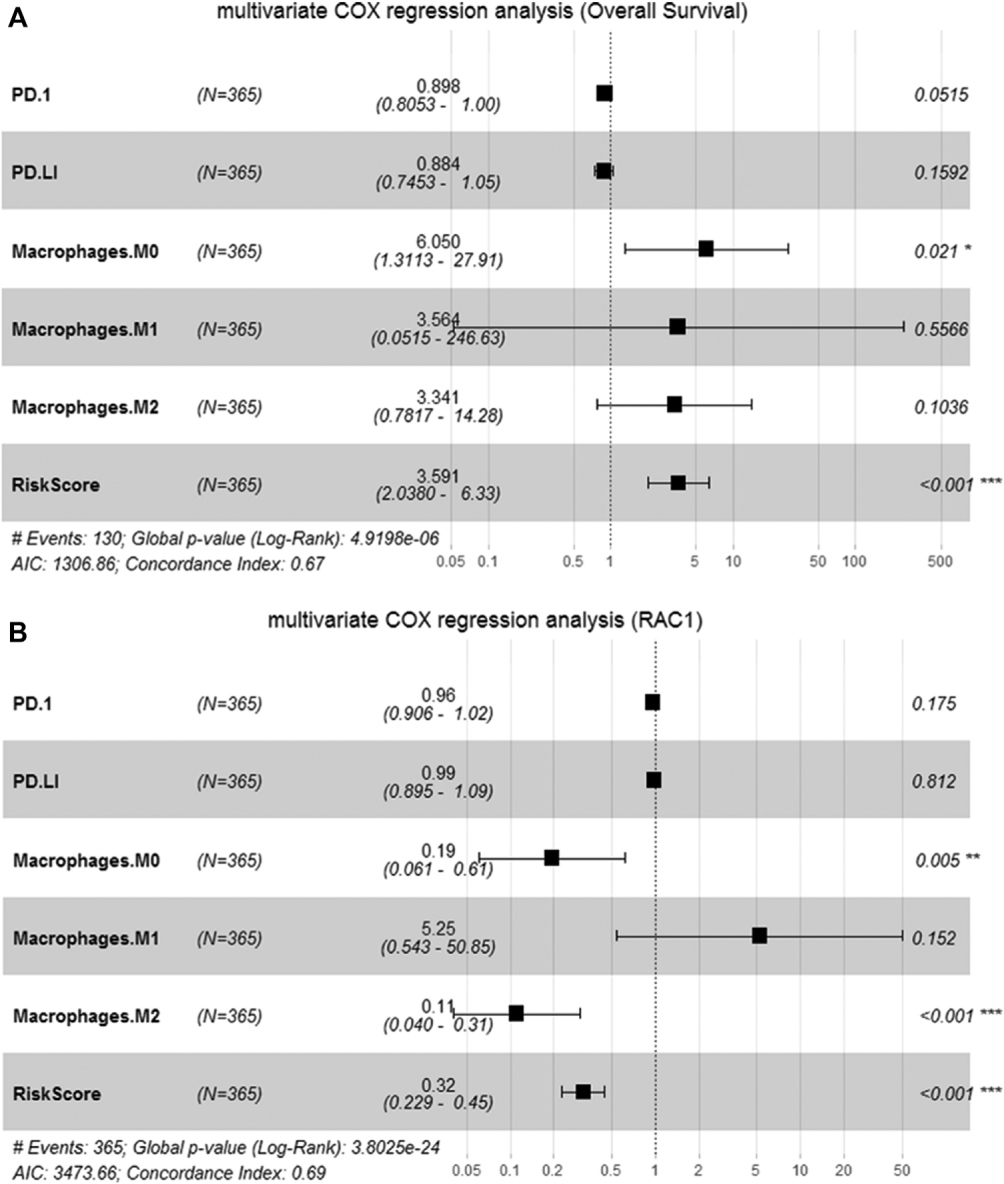
Identification of candidate features. **(A)** Multivariate Cox regression to determine the relationship between candidate features and the overall survival of HCC; **(B)** Multivariate Cox regression to determine the relationship between candidate features and RAC1.

We perform multivariate Cox analysis on the immune and risk characteristics obtained by the algorithm above. M0 macrophage (HR = 6.050, 95% CI = 1.3113–27.91, *p* = 0.021) and risk score (HR = 3.591, 95% CI = 2.038–6.33, *p* < 0.001) were significantly correlated with the survival (Figure 7A). In addition, M0 macrophage (HR = 0.19, 95% CI = 0.061–0.61, *p* = 0.005), macrophage M2 (HR = 0.11, 95% CI = 0.040–0.31, *p* < 0.001) and risk score (HR = 0.32, 95% CI = 0.229–0.45, *p* < 0.001) was significantly correlated with RAC1 (Figure 7B). Therefore, the M0 macrophage and risk score were determined as prognostic factors.

## Discussion

The RAC1 is located on human chromosome 7p22. The RAC1 gene promoter is rich in GC bases and has the characteristics of a housekeeping gene (Payapilly and Malliri, 2018). Its encoded product is an essential member of the small G protein Rho family. RAC1 was widely expressed in various tissues of the body (del Pozo et al., 2004), and the GDP-bound form and GTP-bound form are converted to each other by binding or hydrolyzing GTP nucleotides (Heasman and Ridley, 2008).

Many studies have found that the expression of RAC1 is significantly increased in liver cancer (Lou et al., 2018). For instance, Sharda et al. have shown that stretching liver cancer cells significantly increases RAC1 expression in HCC and cholangiocarcinoma cell lines (Yadav et al., 2020). Furthermore, we found that RAC1 expression has a significant impact on the prognosis of HCC and the relevant immune genes from the risk score for double verification. The results explain the accuracy of RAC1 expression for clinical prediction. In tumor tissues, the active form of RAC1 is involved in regulating the movement of tumor cells and affecting tumor growth by regulating the filopodia and membrane shrinkage (del Pozo et al., 2004). In addition, RAC1 also inhibits tumor cell apoptosis by increasing intracellular superoxide anions (Pervaiz et al., 2001), and plays a critical role in tumor development.

In the study, TCGA and GSE76427 data were used to find that the RAC1 expression in cancer tissues was significantly higher than that of adjacent liver tissues, consistent with the study of Li et al. (2016). It suggests that RAC1 may be involved in the occurrence and development of HCC. MiR-142-3p targeted RAC1 to inhibit the migration and invasion of liver cancer cells (Wu et al., 2011). Survival and time-dependent ROC results confirmed that RAC1 has a good prognostic performance. And the expression of RAC1 is significantly related to T stage, grade and stage. However, univariate and multivariate Cox results confirm that RAC1 is an independent factor of HCC that is not affected by clinical factors. Bayo et al. (2020) also confirmed that RAC1 is an independent target for the new treatment of HCC. 1D-142 targeted inhibition of RAC1 can produce a powerful anti-tumor effect in highly proliferative HCC. Subsequently, we used WGCNA and LASSO algorithms to identify 11 IRGs signatures based on differentially expressed genes. The signature demonstrated a good prognostic performance. Therefore, the new RAC1 marker can be used as a potential prognostic biomarker for liver cancer.

We applied the CIBERSORT algorithm to assess the immune cell infiltration to adjust to the heterogeneity of the HCC microenvironment. The entire macrophage population has a high abundance and apparent abnormal infiltration (M0 M1, M2 macrophage). Also, PD-I and PD-L1 have a strong relationship with macrophages, which is in line with Liu et al. (2018), who discovered that macrophage PD-L1 expression was positively associated with the patient’s overall survival. M0 macrophages in tumors prevent T cells from attacking tumor cells and secrete growth factors that encourage tumor angiogenesis (Vinnakota et al., 2017). The antigen presentation mechanism triggers a Th1 immune response, an essential part of macrophages M1’s anti-tumor effect. And elevated macrophages M1 secrete various pro-inflammatory factors to attract and activate T cells in the early stages of cancer (Aras and Zaidi, 2017).

M0 macrophages play an important role in the occurrence and development of various tumors (Jiang et al., 2020). Analysis based on the expression profile of hypoxia-related genes and clinical information showed that the macrophage M0 cells of HCC patients in the high-risk group were significantly higher than those in the low-risk group. The abundance of M0 macrophages in patients with endometrial cancer in the high-risk group increased significantly (Liu et al., 2020). An immune prognostic signature was constructed based on the TP53 status, and the M0 penetration of macrophages in high-risk gastric cancer patients increased (Nie et al., 2020). Tumors with an increased number of M0 macrophages are related to the poor prognosis of LUAD in the early clinical stage (Liu et al., 2017). Our previous research also found that the abundance of macrophages M0, M1 and M2 all changed drastically during the process of canceration.

On the other hand, M2 macrophages triggered by IL-4 and IL-13 are often used to facilitate cancer progression. It regulates the immune response by secreting IL-10 or TGF-β (Ma et al., 2016). It also secretes MMP to aid tumor cells to achieve metastasis (Vinnakota et al., 2017). Consequently, macrophage infiltration change may be a critical event in the pathogenesis of liver cancer.

We created a prognostic signature that improved the prognostic prediction of HCC patients. The risk score distinguishes between low- and high-risk patients, and the latter shown a poor prognosis (*p* < 0.001). At the same time, the verification study supports the robustness of the prognostic model (*p* = 0.029). Several prognostic models have been published so far. Chen et al. (2019) and Wang et al. (2018) developed a 4- and a 6-gene prognostic model, respectively, similar to ours. However, none of these studies includes a quantitative evaluation of the prognostic model’s predictive survival capacity.

We have previously determined prognostic targets for liver cancer through bioinformatics methods (Yang et al., 2021; Xu et al., 2022). The study further implemented a detailed analysis of transcriptome data to investigate the role of RAC1-induced immune imbalance of HCC. Our research has some drawbacks, including that RAC1 markers have an independent prognosis in liver cancer. On the one side, the study only investigated RAC1-relevant DEGs, and the prognostic markers it used do not represent the HCC genome-wide transcription profile. On the other hand, our findings may need to be confirmed in a large number of clinical trials.

## Conclusion

In summary, we revealed RAC1 correlated with M0 macrophage and the risk score to predict the survival of HCC patients through a comprehensive application of WGCNA, LASSO and SVM. We applied the SVM-based deconvolution algorithm CIBERSORT and WGCNA to assess the fluctuation of the immune microenvironment and found 838 IRGs relevant to macrophage infiltration. Finally, we used LASSO to establish a novel 11-gene signature with a good prognostic performance. The macrophages’ abnormal infiltration driven by RAC1 may serve as an essential immune event in carcinogenesis. Therefore, RAC1 may be a possible important marker for HCC immunotherapy.

## Data Availability

The original contributions presented in the study are included in the article/supplementary material, further inquiries can be directed to the corresponding authors.
